# Transsulfuration Pathway Products and H_2_S-Donors in Hyperhomocysteinemia: Potential Strategies Beyond Folic Acid

**DOI:** 10.3390/ijms26136430

**Published:** 2025-07-03

**Authors:** Lorenzo Flori, Sara Veneziano, Alma Martelli, Eugenia Piragine, Vincenzo Calderone

**Affiliations:** 1 Department of Pharmacy, University of Pisa, via Bonanno Pisano 6, 56126 Pisa, Italy; lorenzo.flori@farm.unipi.it (L.F.); sara.veneziano@phd.unipi.it (S.V.); alma.martelli@unipi.it (A.M.); vincenzo.calderone@unipi.it (V.C.); 2 Interdepartmental Research Center “Nutraceuticals and Food for Health”, University of Pisa, via Del Borghetto 80, 56124 Pisa, Italy; 3 Interdepartmental Research Center “Biology and Pathology of Ageing”, University of Pisa, via Risorgimento 36, 56126 Pisa, Italy

**Keywords:** hydrogen sulfide, sulfur compounds, transsulfuration pathway, homocysteine, hyperhomocysteinemia

## Abstract

The transsulfuration pathway plays a central role in the regulation of sulfur metabolism and contributes to the maintenance of cellular homeostasis. Starting from homocysteine, a sulfur-containing amino acid derived from methionine via the methionine cycle, this metabolic pathway supports the biosynthesis of cysteine and other downstream products, such as taurine, serine, reduced glutathione and the gasotransmitter hydrogen sulfide (H_2_S). The most common disruption of this pathway leads to hyperhomocysteinemia (HHcy), a well-known risk factor for the development of cardiometabolic diseases and other pathological conditions. In this context, identifying effective pharmacological strategies is crucial. Based on both preclinical and clinical evidence, this review provides an updated overview on the role of folates in restoring transsulfuration balance in HHcy and explores the potential effects of downstream products (such as serine, taurine, and precursors of glutathione) under HHcy conditions. Finally, it examines the pharmacological properties of H_2_S-donors in cultured cells exposed to HHcy and in animal models of HHcy. This summary of the literature offers new perspectives for the treatment of HHcy and the prevention of its associated multiorgan complications.

## 1. Introduction

The transsulfuration pathway is a metabolic route that plays a central role in regulating sulfur metabolism and cellular function. Starting from the amino acid homocysteine (Hcy), it supports the biosynthesis of cysteine (Cys) and downstream products, such as taurine, serine, and reduced glutathione (GSH). During these biotransformation reactions, the gasotransmitter hydrogen sulfide (H_2_S) is also generated [1]. Any imbalance in the transsulfuration pathway can lead to the development of various pathological conditions, such as atherosclerosis and hypertension [2,3]. In addition, disruption of this pathway can also contribute to the onset and progression of cardiometabolic disorders (e.g., type 2 diabetes—T2D—and overweight/obesity) [4,5]. Although the causal relationship between disease development and disrupted sulfur metabolism remains to be fully elucidated, the critical role of transsulfuration pathway alterations in different pathological states is well established.

One of the most common consequences of alterations in the transsulfuration pathway is hyperhomocysteinemia (HHcy), a well-known risk factor and potential hallmark of cardiometabolic diseases [6,7,8]. This review describes the pathophysiological relevance of the transsulfuration pathway and discusses the pharmacological properties of both folate cycle players and transsulfuration products, aiming to propose new options for the prevention and treatment of multiple disorders through the restoration of sulfur metabolism.

### 1.1. Transsulfuration Pathway

The transsulfuration pathway starts with Hcy, a sulfur-containing amino acid derived from methionine (Met) through the Met cycle. This three-step process involves the conversion of dietary Met—primarily found in eggs, meat, and legumes—into S-adenosyl Met (SAM), then into S-adenosyl Hcy (SAH) and, finally, into Hcy [1]. The Met cycle is closely regulated by the folate cycle, in which folic acid is first converted into tetrahydrofolate (THF), then into 5,10-methylene THF and, ultimately, into 5-methyl THF by the enzyme methylenetetrahydrofolate reductase (MTHFR). In particular, methionine synthase (MS) catalyzes the remethylation of Hcy to Met using vitamin B12 (cobalamin) as a co-factor and 5-methyl THF as the methyl donor [9]. Therefore, any imbalance in the folate cycle can disrupt the Met cycle and vice versa. Remethylation of Hcy can also occur through the transfer of a methyl group to produce Met. This reaction is catalyzed by the enzyme betaine-homocysteine methyltransferase (BHMT), which uses betaine as the methyl donor to generate dimethylglycine (DMG) [10].

**Figure 1:**
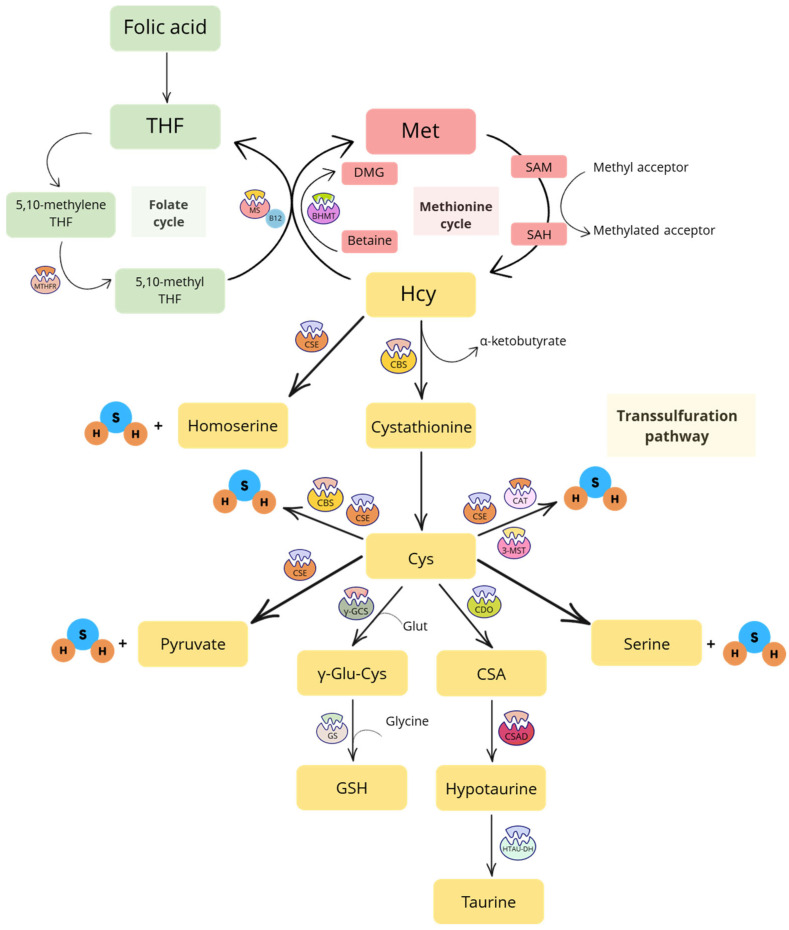
Schematic representation of the folate cycle, methionine cycle and transsulfuration pathway. Abbreviations: 3-MST, 3-mercaptopyruvate sulfurtransferase; BHMT, betaine-homocysteine methyltransferase; CAT, cysteine aminotransferase; CBS, cystathionine β-synthase; CDO, cysteine dioxygenase enzyme; CSA, cysteine sulfinic acid; CSAD, cysteine sulfinic acid decarboxylase; CSE, cystathionine γ-liase; Cys, cysteine; DMG, dimethylglycine; Glut, glutamate; GS, glutathione synthetase; GSH, reduced glutathione; H_2_S, hydrogen sulfide; Hcy, homocysteine; HTAU-DH, hypotaurine dehydrogenase; Met, methionine; MS, methionine synthase; MTHFR, methylenetetrahydrofolate reductase; SAH, S-adenosyl homocysteine; SAM, S-adenosyl methionine; THF, tetrahydrofolate; γ-GCS, γ-glutamyl cysteine synthetase; γ-Glu-Cys, γ-glutamyl cysteine.

The transsulfuration pathway intersects with the Met cycle at Hcy. The enzyme cystathionine β-synthase (CBS) catalyzes the irreversible conversion of Hcy into cystathionine, which is the substrate for the enzyme cystathionine γ-liase (CSE). CSE then converts cystathionine into Cys and α-ketobutyrate. This is the only pathway for the biosynthesis of the amino acid Cys. Once produced, Cys can be further transformed into taurine (via cysteine dioxygenase—CDO—cysteine sulfinic acid decarboxylase—CSAD—and hypotaurine dehydrogenase—HTAU-DH), GSH (via γ-glutamyl cysteine synthetase—γ-GCS—and glutathione synthase—GS), pyruvate, serine and H_2_S (via CBS and CSE enzymes). The gaseous molecule H_2_S can be also derived from the conversion of Hcy into homoserine, catalyzed by CSE [1], and from Cys through the conversion operated by cysteine aminotransferase (CAT) and 3-mercaptopyruvate sulfurtransferase (3-MST) [1] (Figure 1).

Under physiological conditions, the transsulfuration pathway ensures the bioavailability of (i) antioxidant compounds that participate in the regulation of the intracellular redox state (i.e., GSH) [11]; (ii) the non-sulfur amino acid serine, that is involved in many cellular functions, serving as a substrate for glucose, protein, and phospholipid synthesis, and playing a central role in both the folate and Met cycles [12]; (iii) pyruvate, which is the primary “fuel” for the mitochondrial respiratory chain and adenosine triphosphate (ATP) production in these organelles [13]; (iv) taurine, a sulfur-containing amino acid that reduces oxidative stress and inflammation, promotes mitochondrial health, and plays a role in immunomodulation, osmoregulation and calcium homeostasis [14]; and (v) H_2_S, known as a toxic agent with the distinctive smell of rotten eggs until 1996, when Abe and Kimura first described its biosynthesis in mammals [15], marking a new era in sulfur pharmacology. Together with carbon monoxide (CO) and nitric oxide (NO), H_2_S is now recognized as the third endogenous gasotransmitter, which signals through post-translational modifications of proteins (i.e., S-sulfhydation of Cys residues) [16,17]. This process leads to the modulation of enzyme activity (i.e., activation/inhibition), the opening or closing of ion channels, and the regulation of gene and protein expression (i.e., increase or decrease), with subsequent biological effects that range from antioxidant and anti-inflammatory to vasorelaxant, cardioprotective and neuroprotective [18,19,20].

Consistent with the antioxidant and cytoprotective effects of these downstream products, any imbalance in the transsulfuration pathway can disrupt organ and tissue homeostasis. On the contrary, activation of this pathway through CBS stimulation attenuated oxidative stress and prevented programmed cell death, particularly ferroptosis, in mice with sickle cell disease [21]. Moreover, CBS activation contributed to the resistance of tumor cells to ferroptosis [22]. A “strong proof” of the crucial role of the transsulfuration pathway in organ and tissue homeostasis was provided by Mota-Martorell and colleagues in 2021, who demonstrated that both the Met cycle and transsulfuration pathway are up-regulated in centenarians. In fact, these individuals exhibit significantly higher plasma levels of Cys and cystathionine compared to adults and the elderly (non-centenarians) [23].

### 1.2. Hyperhomocysteinemia (HHcy)

**Figure 2:**
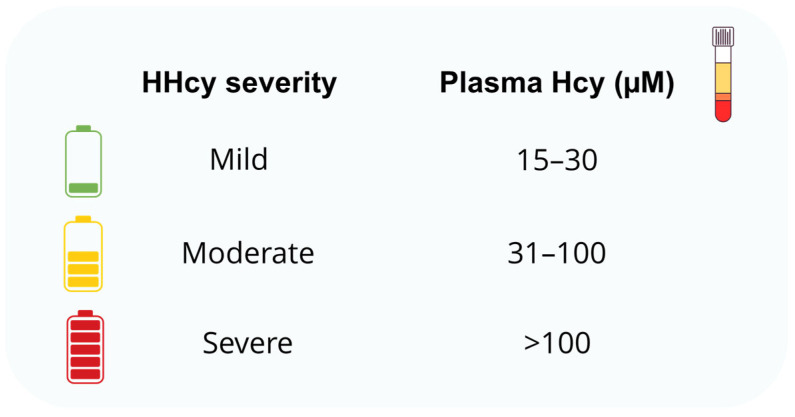
Classification of hyperhomocysteinemia (HHcy) severity based on plasma homocysteine (Hcy) concentrations in humans.

Alterations in the transsulfuration pathway often lead to HHcy, a pathological condition characterized by elevated plasma levels of Hcy (Figure 2) [24].

As previously mentioned, HHcy is a well-established cardiovascular (CV) risk factor. In fact, HHcy contributes to the development of atherosclerosis and related CV complications mainly through mechanisms involving inflammation, altered lipoprotein metabolism, and oxidative stress [25]. Elevated Hcy levels have been found in patients with hypertension [26], heart failure [27], and stroke [28,29]. HHcy is also frequently reported in patients with metabolic disorders, including T2D [5] and overweight/obesity [4,26], suggesting that dysregulation of the transsulfuration pathway may play a role in the onset, progression and/or complications not only of CV diseases but, more broadly, of cardiometabolic disorders [5]. This link was confirmed in a mouse model of diet-induced HHcy, where mice fed a high-Met, low-folate diet for 8 weeks developed glucose intolerance and insulin resistance [30].

As illustrated in Figure 1, Hcy is a central intermediate in both the Met cycle and the transsulfuration pathway, and its levels are tightly regulated to maintain metabolic homeostasis. However, under specific conditions, excessive Hcy can accumulate, leading to HHcy. This may result from diet-induced hypermethioninemia [31], defects in Met or folate metabolism [32], inadequate intake or absorption of vitamin B12 or betaine, or deficiencies/mutations in the enzymes MTHFR and MS—which catalyze the remethylation of Hcy to Met—or BHMT—that remethylates Hcy using betaine as a donor [33]. HHcy may also stem from low THF levels, often due to insufficient folate intake or reduced absorption [32]. Finally, HHcy could result from a deficiency of CBS, which impairs the conversion of Hcy to cystathionine, leading to Hcy accumulation [32,34]. One of the most widely used mouse models of HHcy in preclinical studies is the CBS^+/−^ mouse. However, HHcy can also be induced experimentally by feeding animals a Met-rich diet and/or a low-folate diet [30,35].

The role of insufficient folate or excessive Met consumption in the development of HHcy has been highlighted in various studies, mainly focused on brain health [31]. Dietary supplementation with Met (2 g/kg body weight—BW—daily for 4 weeks) significantly increased plasma Hcy levels and induced both metabolic and histo-morphological changes in the rat hippocampus following global ischemia [36]. Similarly, mice fed a high-Met, low-folate and low-vitamin B12 diet (1.2%, 0.08 mg per kg of diet, and 0.01 mg/kg per kg of diet, respectively, for 1–6 weeks) developed HHcy and, as a consequence, endothelial hyperpermeability in cerebral vessels. These animals exhibited vascular inflammation, endothelial dysfunction, and disruption of the blood–brain barrier, with subsequent neurodegeneration and short-term memory impairment [36].

These findings suggest that HHcy-induced organ and tissue damage may stem primarily from the loss of endothelial barrier integrity and function [37,38,39,40,41]. Disturbances in vascular homeostasis, endothelial dysfunction due to reduced NO bioavailability, and increased endothelial permeability can promote the spread of inflammatory mediators, reactive oxygen species (ROS) and Hcy itself into surrounding tissues. This generates a “vicious cycle” that exacerbates inflammation and contributes to progressive multiorgan damage [42,43]. Furthermore, induced HHcy is known to accelerate atherosclerosis in ApoE^−/−^ mice, which are genetically predisposed to developing this pathological condition [44]. HHcy can increase total cholesterol, triglycerides, and low-density lipoprotein (LDL) cholesterol, while decreasing high-density lipoprotein (HDL) cholesterol in these mice, accelerating the development and instability of atherosclerotic plaques [45]. Additionally, both clinical and preclinical data indicate that HHcy might be a potential etiological factor in chronic heart failure (CHF) [46]. Indeed, high Met levels in a high-fat diet can induce HHcy, leading to cardiac remodeling and the development of heart failure with preserved ejection fraction (HFpEF) [47].

Regardless of its underlying cause, any imbalance in the transsulfuration pathway can impair the bioavailability of Cys and, therefore, the production of downstream sulfur-containing mediators (e.g., GSH, taurine, and H_2_S). For instance, GSH deficiency has been detected in mice brain slices [48] and in cultured hepatocytes [49] following treatment with inhibitors of the CSE enzyme. Reduced expression of CSE was found in a rat model of kidney ischemia-reperfusion, in which elevated plasma Hcy levels were accompanied by decreased levels of GSH in both liver and plasma [50]. An inverse relationship between intracellular Cys and GSH concentrations and plasma Hcy levels has been also reported in rats fed a high-fat diet [51] and in aged individuals [52].

Increasing evidence indicates that HHcy may dysregulate epigenetic mechanisms, particularly through changes in DNA methylation and histone N-homocysteinylation. For instance, HHcy promotes the intracellular accumulation of SAH and reduces the SAM/SAH ratio, ultimately leading to global DNA hypomethylation [53]. This imbalance can affect the expression of genes involved in the transsulfuration pathway, including *CBS* and *CSE*. Notably, SAM not only serves as the main methyl donor for DNA and histone methylation but also acts as an allosteric activator of CBS via binding to its C-terminal regulatory domain, thereby enhancing H_2_S production [54]. Thus, SAM depletion in HHcy may simultaneously impair epigenetic regulation and enzymatic activation within the transsulfuration pathway [20]. Together, these findings suggest a dual epigenetic and metabolic mechanism linking HHcy to transsulfuration dysfunction, although further studies are warranted to confirm this hypothesis.

Regarding taurine, the impact of transsulfuration pathway imbalance on this sulfur-containing amino acid is less defined. However, a significant inverse correlation between plasma levels of taurine and Hcy was observed in middle-aged women, suggesting a possible relationship between these two sulfur-containing amino acids [55].

The imbalance between Hcy and H_2_S has been more extensively investigated [56]. Reduced plasma levels of the gasotransmitter have been observed in CBS^+/−^ mice [57]. Similarly, intraperitoneal administration of DL-Hcy (0.3 mmol/g BW in the first week, increased to 0.6 mmol/g BW by the third week, twice daily for 3 weeks) significantly decreased H_2_S levels in the rat myocardium [58]. Reduced biosynthesis of H_2_S was also detected in human retinal endothelial cells co-exposed to D-glucose (20 mM) and Hcy (100 µM), as well as in the retina of mice with HHcy and streptozotocin-induced T2D [35]. Finally, dietary Met supplementation in female rats (7.7 g/kg per kg of diet), 3 weeks prior to and during pregnancy, led to a significant reduction in H_2_S levels in the brains of the offspring [59], while treatment of mice with Hcy (0.03 µmol/g BW, twice a day for 30 days, subcutaneously) decreased H_2_S production in the brain [60]. This evidence corroborates the hypothesis of a mutual regulatory relationship between H_2_S and Hcy [61] and, at least in part, may help explain the observed decline in H_2_S levels in patients with age-related, and potentially HHcy-associated, disorders [62]. Intriguingly, CBS and CSE are also secreted by microvascular endothelial cells and hepatocytes into the bloodstream, supporting the presence of an “extracellular transsulfuration system” that produces H_2_S starting from Hcy and protects the endothelium from Hcy-induced oxidative stress “from the outside” [63].

**Figure 3:**
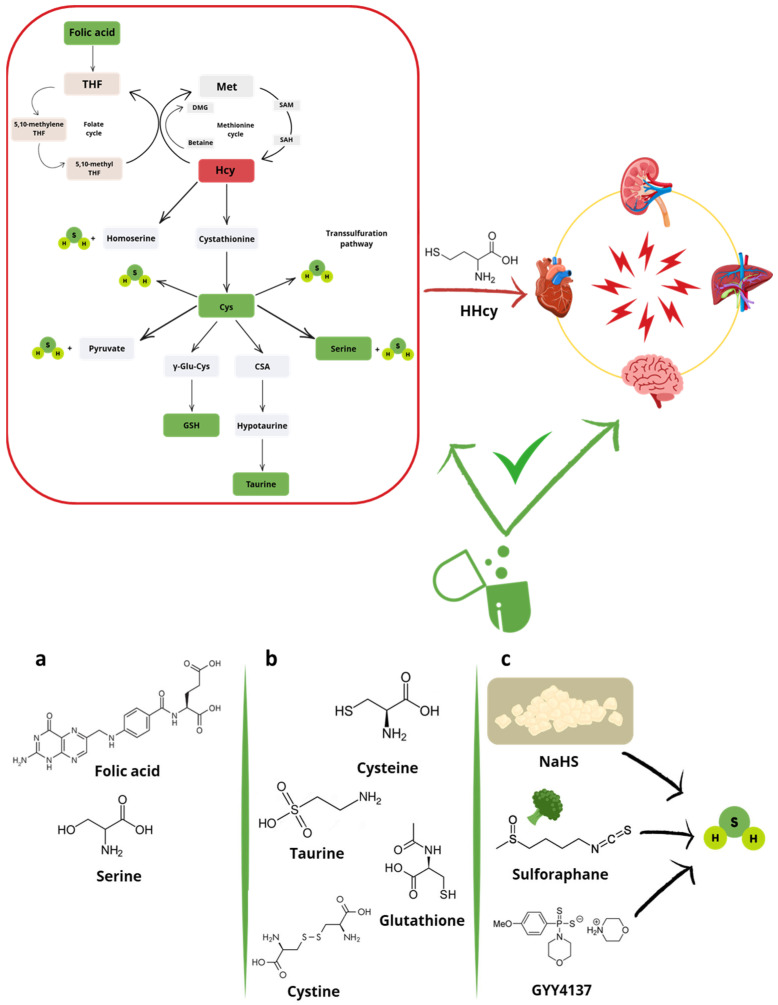
Hyperhomocysteinemia, the most common alteration of the transsulfuration pathway (red plot), contributes to multiorgan damage. This imbalance can potentially be restored (green plot) through supplementation/treatment with (**a**) folic acid and non-sulfur amino acids (i.e., serine), (**b**) sulfur-containing products of the transsulfuration pathway (i.e., cysteine, taurine, glutathione, or cystine), and (**c**) hydrogen sulfide (H_2_S)-donors. Abbreviations: CSA, cysteine sulfinic acid; Cys, cysteine; DMG, dimethylglycine; GSH, reduced glutathione; Hcy, homocysteine; HHcy, hyperhomocysteinemia; Met, methionine; SAH, S-adenosyl homocysteine; SAM, S-adenosyl methionine; THF, tetrahydrofolate; γ-Glu-Cys, γ-glutamyl cysteine.

In conclusion, the main hypothesis of this review is that restoring the balance of the transsulfuration pathway through supplementation with folic acid or downstream products—namely taurine, GSH, and H_2_S—(Figure 3) may reduce HHcy and, consequently, mitigate the associated oxidative stress. Accordingly, folic acid promotes indirect antioxidant effects by lowering Hcy levels [64], thereby rebalancing the transsulfuration pathway and enhancing both total antioxidant capacity and serum GSH concentrations [65]. However, taurine, H_2_S, and the precursors of GSH may offer additional effects beyond the mere restoration of physiological concentrations. Taurine and the GSH precursor N-acetylcysteine (NAC) exhibit antioxidant effects through direct scavenging activity of both radical and non-radical species [66,67]. Taurine also attenuates oxidative stress by preserving mitochondrial electron transport chain function, stabilizing biological membranes, inhibiting ROS-generating enzymes, such as xanthine oxidase (XO) and NADPH oxidase (NOX), and by promoting nuclear translocation of the antioxidant transcription factor nuclear factor erythroid 2-related factor 2 (Nrf2) and inhibiting that of the nuclear factor kappa-light-chain-enhancer of activated B cells (NF-κB) [66]. NAC, instead, supports the biosynthesis of GSH which, in turn, directly reacts with oxidants and serves as a substrate or cofactor for detoxifying enzymes [67]. Finally, the downstream product H_2_S acts as a reducing agent that directly neutralizes ROS and reactive nitrogen species (RNS) [68]. Beyond this direct scavenging activity, H_2_S exerts more “sophisticated” antioxidant effects. A key mechanism involves the translocation of the antioxidant transcription factor Nrf2 from the cytoplasm to the nucleus, where it binds the antioxidant response element (ARE) and enhances the expression of antioxidant genes, such as superoxide dismutase (*SOD*), glutathione peroxidase (*GPx*), and heme oxygenase-1 (*HO-1*). Specifically, under oxidative stress conditions, such as those induced by HHcy, H_2_S promotes dissociation of Nrf2 from its cytoplasmic inhibitor Kelch-like ECH-associated protein 1 (Keap1) via S-sulfhydation of Cys residues of Keap1, particularly Cys151 [69]. Moreover, H_2_S S-sulfhydates sirtuin isoform 1 (SIRT1) at Cys371, Cys374, Cys395 and Cys398, thereby enhancing its deacetylase activity and further amplifying the Nrf2-mediated antioxidant response [70]. Another mechanism underlying the antioxidant and anti-inflammatory effects of H_2_S is the inactivation of the transcription factor NF-κB. Through S-sulfhydation of the p65 subunit of NF-κB at Cys38, H_2_S prevents its nuclear translocation, thus suppressing the transcription of pro-inflammatory and redox-related genes [71,72].

Besides oxidative stress, emerging evidence suggests that ferroptosis, an iron-dependent form of cell death driven by lipid peroxidation, may represent an additional downstream consequence of HHcy-induced metabolic imbalance. Mechanistically, HHcy has been shown to impair mitochondrial electron transport chain efficiency, increase ROS and iron levels, and deplete intracellular GSH, thereby predisposing cells to ferroptosis [73,74]. In particular, HHcy disrupts the anti-ferroptotic cystine/glutamate antiporter-GSH-GPx4 (Xc^−^-GSH-GPx4) axis [75,76] by reducing Cys availability and impairing GPx4 activity, resulting in lipid peroxidation and ferroptotic cell death [73]. H_2_S has recently emerged as a potential modulator of ferroptosis. One mechanism involves the S-sulfhydation of OTU domain-containing ubiquitin aldehyde-binding protein 1 (OTUB1) at Cys91, which stabilizes the Xc^−^ light chain subunit (xCT), thereby promoting cystine uptake and maintaining GSH biosynthesis [77]. Moreover, H_2_S may exert anti-ferroptotic effects by preserving mitochondrial function. In this regard, Wang and colleagues showed that exogenous H_2_S restores the expression of GPx4 and mitochondrial cysteine desulfurase (NFS1) via S-sulfhydation of optic atrophy 3 (OPA3), a key regulator of mitochondrial homeostasis [78]. This suggests that H_2_S may contribute to ferroptosis resistance through both cytoplasmic and mitochondrial pathways. Similarly, Cys depletion can trigger ferroptosis, mainly due to impaired GSH biosynthesis and defective Fe/S cluster biogenesis, which result in an iron starvation response, increased iron uptake, and subsequent ferroptotic cell death [79,80].

## 2. Players of the Folate Cycle

The ability of folic acid, vitamin B12 and B6 supplementation to reduce serum Hcy levels is well known and described in the literature. Daily supplementation with 0.5–5.0 mg of folic acid typically reduces plasma Hcy levels to about 25%. This containment of Hcy levels promotes several benefits for systems/tissues involved in pathological conditions such as CV, renal, hepatic and cerebral diseases [81].

The effects of folate supplementation in HHcy-associated CV diseases have been described extensively using both in vitro and in vivo preclinical models.

The incubation of folic acid in human umbilical vein endothelial cells (HUVECs) for 48 h in a concentration range of 0–1000 nM, in combination with Hcy 1 mM or with the vehicle for the first 24 h, led to positive effects on cell viability and reduced apoptosis in a concentration-dependent manner. The authors underlined that this could be mediated by the reduction in caspase-3/7 activity, by the increase in the B-cell lymphoma 2/BCL-2-like protein 4 (BCL2/BAX) ratio and by the reduction in tumor protein 53 (TP53), caspase-3 (CASP3) and caspase-8 (CASP8) expression [82]. Overlapping positive effects on the endothelial component were also described by Zhang and colleagues demonstrating that the incubation of folic acid (5–10 nM for 12 h) under Hcy stimulation (0.1 μM for 12 h) significantly increased NO production and the levels of tetrahydrobiopterin (BH4), analyzed as a cofactor of endothelial nitric oxide synthase (eNOS) functionality [83].

Turning to the evidence collected in in vivo models, rats fed a diet containing 3 g of Hcy per kg of diet for 2 weeks and subsequently supplemented with 8 mg of folic acid per kg of diet for 8 weeks, showed a significant containment of the increase in serum Hcy levels and an associated significant reduction in the incidence of vascular damage. The authors suggested that folic acid supplementation may reduce the endothelial-damaging effects of HHcy [84]. Furthermore, a daily gavage of 0.4 mg/kg folic acid (6 weeks of treatment starting after 4 weeks of Hcy intraperitoneal injection) in spontaneously hypertensive rats (SHRs) was sufficient to reverse interstitial and perivascular collagen deposition in cardiomyocytes and diastolic dysfunction exacerbated by HHcy (2% DL-Hcy, 5 mL/kg by intraperitoneal injection twice daily). These effects were associated with the translocation of the antioxidant transcription factor Nrf2 from the cytoplasm to the nucleus, and with the increased expression of *HO-1*. Administration of a higher dose of folic acid (4 mg/kg daily gavage for 6 weeks starting after 4 weeks of Hcy intraperitoneal injection), tested under the same experimental conditions, did not promote improvements compared to the lowest concentration tested [85].

Moving on to the clinical evidence, folate supplementation (0.5–5 mg daily) effectiveness in containing HHcy emerged from some meta-analyses evaluating its efficacy both with and without vitamin B6 (mean 16.5 mg daily) and B12 (mean 0.5 mg daily) supplementation. The results confirmed that a significant effect of folates in the reduction in serum Hcy levels is further enhanced in conditions of HHcy associated with reduced serum folate concentrations at baseline. Furthermore, the combined supplementation of vitamin B12 favored an additional reduction of circulating Hcy levels of about 7%, while the combined supplementation of vitamin B6 did not show a significant additional effect [86]. Subsequent studies focused on the importance of the intake of vitamins involved in the folate cycle highlighted how the intake of folates (50–700 μg/day) and vitamin B6 (0.5–5 mg/day) or its diet supplementation was associated with a reduced risk of coronary heart disease and CV disease after a follow-up period ranging between 8 and 16 years [87,88,89]. A 2-year, randomized, placebo-controlled, double-blind study conducted on 186 patients with end-stage renal disease demonstrated that oral folic acid supplementation (10 mg, 3 times weekly, immediately after each dialysis session) normalized blood Hcy levels in 92.3% of patients but did not reduce the incidence of CV events compared with the placebo group. However, ultrasound examination of the common carotid arteries performed 24 months after treatment showed a significant reduction in intima-media wall thickness with folate supplementation [90].

In general, experimental evidence is in line with the positive effects of folic acid supplementation in reducing serum Hcy levels in conditions of HHcy associated with cardiometabolic diseases. In particular, a meta-analysis of observational studies suggested that lowering Hcy by 3–4 μmol/L results in an approximately 30–40% reduction in vascular disease [81]. Another meta-analysis including 30 randomized controlled trials involving 82.334 participants indicated a 10% reduction in stroke risk and a 4% reduction in overall CV risk with folic acid supplementation (0.5–40 mg/day) analyzed with or without vitamin B6 or B12 supplementation. Greater CV benefit was observed among participants with lower plasma folate levels and no preexisting CV disease, as well as in patients with greater reductions in Hcy levels overall [91].

## 3. Products of the Transsulfuration Pathway

Among the main products of the transsulfuration pathway there are taurine and GSH. Although the correlation between cardiometabolic pathologies, alterations of the transsulfuration pathway characterized by HHcy, and reduced levels of GSH is widely studied and described, scientific evidence on the effects of exogenous treatment with GSH aimed at restoring its physiological levels is rather lacking. On the other hand, administration of Cys, cystine, and NAC, as precursors of GSH, is exhaustively represented by experimental evidence that underlines their positive effects in containing HHcy. Finally, serine and taurine are also subjects of several studies aimed at evaluating the cardiometabolic effects of their exogenous supplementation under HHcy states.

### 3.1. Central Products (Cys, Cystine and Serine) and Synthetic Derivatives (NAC)

Preclinical experimental evidence has highlighted the importance of some amino acids involved in the transsulfuration pathway and their derivatives in the regulation of plasma and tissue Hcy levels. For example, Cys administration (diets enriched with 0.3% or 0.6% for 14 days) significantly suppressed HHcy induced by a low-protein diet (10% casein) in rats [92]. Furthermore, Cys supplementation (1.5 mg/mL in drinking water for 1 week) normalized hepatic CDO protein levels in a mice model of homocystinuria (HCU) [93].

In vivo experimental models have demonstrated the efficacy of serine in suppressing Met-induced HHcy. Serine, either supplemented to a 0.5, 1, or 2% Met diet for 10 days or injected intraperitoneally with Met (100, 200, 300, or 500 mg/kg), was able to significantly reduce Hcy levels in rats. The authors of this study suggested that this effect could be due to stimulation of cystathionine production by providing serine as an alternative substrate for its synthesis [94]. Moreover, a randomized crossover trial conducted on 24 healthy men pointed out that serine (60.6 mg/kg) and cystine (12.3 mg/kg), supplemented through four meals on 3 consecutive days separated by 1 week, attenuated the increase in plasma Hcy induced by a low-protein diet supplemented with Met (30 mg/kg) [95].

Several preclinical studies have evaluated the effects of NAC in HHcy conditions. NAC (1 g/kg/day for 6 weeks) was capable of decreasing serum Hcy, hepatic and renal ROS, renal SOD and GPx activities, and malondialdehyde (MDA) levels in rats with HHcy induced by homocysteine thiolactone (HcyT; 500 mg/kg/day for 6 weeks) [96]. Moreover, NAC (5 mM for 48 h) counteracted the morphofunctional alterations induced by HHcy (800 μM for 48 h) in the vascular endothelium (i.e., in HUVEC cells) [97]. Two double-blind, placebo-controlled studies in drug-naïve middle-aged men, stratified by smokers and nonsmokers, the first one in a hyperlipidemic group (n = 40) and the second one in a normolipidemic group (n = 42), demonstrated the effect of NAC administration on Hcy regulation. Four weeks of oral NAC treatment (1.8 g/day) significantly reduced plasma Hcy concentrations and lowered systolic blood pressure, suggesting that increased oral Cys intake may be considered for the prevention of vascular diseases associated with HHcy [98]. HHcy is an independent CV risk factor that is strongly affected by renal function and clearance. It is not surprising that the overall HHcy incidence rate of around 7% moves to 85% in patients with chronic renal disease. A baseline condition of HHcy in this clinical population was confirmed in 60 patients with end-stage renal failure. They were randomized to receive a continuous intravenous infusion of NAC (5 g diluted in 5% glucose solution) or placebo (5% glucose solution) during a 4-h hemodialysis session. The results showed a significant reduction in plasma Hcy levels after hemodialysis in the group receiving NAC compared to baseline conditions, and this reduction was significantly higher than that observed in the placebo group. The clear improvement in Hcy levels promoted by NAC was also accompanied by a significant reduction in blood pressure and pulse pressure [99]. These results confirmed the efficacy of NAC in patients with end-stage renal failure reported in previous studies, where a reduction in Hcy levels observed in patients treated with NAC was associated with an improvement in endothelial function [100].

### 3.2. Final Products (Taurine)

In preclinical studies, taurine (10 mM for 30 min) was able to restore the secretion and expression of extracellular superoxide dismutase (EC-SOD), a glycoprotein secreted by vascular smooth muscle cells (VSMCs) that protects the vascular wall from oxidative stress, ameliorating endoplasmic reticulum (ER) stress induced by Hcy (5 mM for 12 h) in rat VSMCs [101]. Taurine (5, 10, and 20 mM for 12 h) reduced the release of lactate dehydrogenase (LDH) and ROS, and increased mitochondria Mn-superoxide dismutase (Mn-SOD) and catalase activity in a concentration-dependent manner in VSMCs co-administered with Hcy (0.5 mM for 12 h) [102]. The reduction of ER stress promoted by taurine (40 mM for 60 min) was also associated with reduced apoptosis in rat cardiomyoblasts (H9c2 cells) exposed to HHcy (1 mM for 24 h) [103].

As detailed by Chang and colleagues, taurine reduced the inhibition of Ca^2+^ uptake induced by Hcy (0.5 mM) in rat mitochondria. Furthermore, taurine held the inhibitory effect of Hcy on Ca^2+^-ATPase activity, with a biphasic action: in the absence of Hcy, at concentrations of 5 and 10 µM, taurine increased Ca^2+^-ATPase activity, but the concentration of 20 µM inhibited Ca^2+^-ATPase activity. Taurine also prevented the generation of hydrogen peroxide and superoxide anions caused by Hcy [104]. Furthermore, taurine supplementation (1% L-taurine in tap water for 6 weeks) improved the functional and structural damage of myocardial mitochondria in rats with Met-induced HHcy (1% DL-Met mixed in normal chow diet for 6 weeks) by reducing ROS production and improving Ca^2+^ fluxes. Taurine also inhibited the products of lipid peroxidation induced by HHcy [105].

Regarding the liver-affecting HHcy condition, some experimental evidence has shown that taurine has protective effects on HHcy-induced hepatotoxicity by reducing oxidative and nitrosative stress, apoptosis and hepatic necrosis. The administration of taurine (1.5% *w*/*v* in drinking water) in rats fed with a Met-rich diet (2% *w*/*w*) for 6 months reduced the activity of serum alanine and aspartate transaminases, hepatic lipid peroxide levels and nitrotyrosine formation, without any change in serum Hcy levels. Decreased BAX expression, increased BCL2 expression, decreased apoptotic cells, and improved histopathological findings were observed in rat livers [106].

In a mice model of CBS-deficient HCU, a severe condition in which deficiency of CBS hinders the triggering of the transsulfuration pathway causing an accumulation of Hcy and a consequent reduction in downstream products such as taurine, Cys, and GSH, taurine supplementation promoted significative improvements. Specifically, taurine supplementation (20 mg/mL in drinking water for 1 week) normalized hepatic expression levels of (i) CDO, the enzyme responsible for the conversion of Cys to cysteinsulfinate, (ii) CSAD, which catalyzes the conversion of cysteinsulfinate to hypotaurine, and (iii) the cytoplasmic isoform of glutamic oxaloacetic transaminase 1 (GOT1) [93,107].

Finally, the results obtained by Van Hove and colleagues are of considerable interest. In a phase 1/2 clinical trial, the pharmacokinetic profile, safety, and effects of taurine on oxidative stress, inflammation, and vascular dysfunction were evaluated in 14 patients (8–50 years; eight males, six females) with inherited CBS-deficient HCU. Taurine 75 mg/kg (max 5 g) administered orally twice daily for 4 days was safe and well-tolerated in no preexisting hypertriglyceridemia condition. Taurine pharmacokinetics were relatively fast in plasma with a slow accumulation in a third compartment. Taurine was unable to positively modulate parameters of oxidative stress and inflammation, probably due to the short treatment duration, but improved brachial artery flow-mediated dilation (FMD), a measure of endothelial function, in the subset of patients with FMD values < 10% and in individuals with Hcy levels > 125 μM at baseline [108].

The pharmacological effects of both players of the folate cycle and products of the transsulfuration pathway under HHcy conditions are summarized in Table 1.

## 4. H_2_S-Donors

In recent years, the potential rebalancing effect of exogenous H_2_S on the transsulfuration pathway under metabolic alterations, including HHcy, has been proposed. Since the direct use of H_2_S is not feasible due to its gaseous nature, many studies have focused on sulfur salts, which are valuable for experimental purposes due to their low cost and wide availability. Among these, sodium hydrosulfide (NaHS), sodium sulfide (Na_2_S), and calcium sulfide (CaS) are the most commonly used. In aqueous solution, these salts rapidly dissociate to release S^2−^ and/or HS^−^ ions which, in turn, are protonated into H_2_S, particularly under acidic pH conditions but even in near-neutral conditions (given the very weak acidic properties of H_2_S). In contrast, “slow” H_2_S-donors, including the synthetic compounds GYY4137 and AP-39, as well as the natural isothiocyanate sulforaphane, provide a slow and prolonged release of the gaseous molecule [109,110,111,112]. Specifically, GYY4137 undergoes spontaneous hydrolytic decomposition in aqueous solution at 37 °C leading to low but sustained concentrations of H_2_S [113]. AP-39 is a mitochondria-targeted “slow” H_2_S-donor [114], and isothiocyanates slowly release the gaseous molecule via nucleophilic reaction with Cys followed by spontaneous cyclization of the intermediate product [115]. The rapid release of H_2_S from “fast” H_2_S-donors may lead to potential toxic effects, such as excessive blood pressure reduction, due to the hormetic behavior of the gasotransmitter (i.e., beneficial at low concentrations but harmful at higher ones) [116]. Moreover, these compounds often induce only transient therapeutic effects, making precise dose control challenging. These limitations reduce the clinical applicability of fast-releasing H_2_S-donors [117,118]. In contrast, “slow” H_2_S-donors more closely mimic the endogenous biosynthesis of the gaseous molecule, allowing not only controlled but also sustained release. This minimizes the risk of acute toxicity and allows prolonged therapeutic exposure, thereby opening new avenues for drug discovery. Synthetic slow-releasing H_2_S-donors largely differ in their chemical structure and include, among others, isothiocyanates [112,119], thioureas [120,121], thioamides [121], and iminothioethers [111]. Notably, even within the same chemical class, H_2_S-donors are not functionally equivalent. For instance, para-substituted aromatic isothiocyanates (e.g., 4-carboxyphenyl isothiocyanate [122], 4-hydroxybenzyl isothiocyanate [112], and 4-nitrophenyl isothiocyanate [115]) are generally more potent H_2_S-releasing agents than aliphatic isothiocyanates [112,123]. This highlights the need for precise in vitro characterization of each H_2_S-donor before advancing to in vivo models. Another critical limitation is the lack of targeted delivery: most H_2_S-donors distribute systemically, increasing the risk of off-target effects. This challenge could be addressed by developing site-specific delivery strategies and enhancing formulation technologies. Additionally, controlled-release formulations may help sustain therapeutic H_2_S concentrations over time, particularly given the short biological half-life of this gaseous molecule [124,125,126].

In the following sections, the impact of both “fast” and “slow” H_2_S-donors on the restoration of transsulfuration pathway alterations under HHcy will be described. The available experimental evidence will be organized by the organ system. In particular, the cardiovascular system (heart and vessels), brain, kidneys and liver will be treated separately to enhance clarity and to account for organ-specific differences in the regulation of the transsulfuration pathway. Results from in vitro studies have shown that, in the presence of equimolar concentrations of CBS and CSE, CBS is fully activated by SAM, leading to the production of H_2_S at a ratio of 7:3 compared to the H_2_S produced by CSE. Therefore, CBS is the primary source of H_2_S under physiologically relevant substrate concentrations [127]. However, tissue-specific differences in CBS and CSE expression levels must be considered. For example, CBS is the main enzyme responsible for H_2_S production in the brain [15], while CSE plays the leading role in the heart and vessels [128]. In general, CSE is more highly expressed than CBS in peripheral organs, including the liver and kidney, with a ratio of about 60:1 in the liver and about 20:1 in the kidney [129].

Interestingly, the production of H_2_S by CBS and CSE may also be influenced by Hcy levels and is inversely related to its concentration. In fact, increasing Hcy levels in the liver shifts the contribution of H_2_S production more toward CSE. In other words, the higher the hepatic levels of Hcy, the more predominant CSE becomes over CBS in the transsulfuration pathway [129].

### 4.1. Heart and Vessels

About 10 years ago, Nandi and co-workers studied the potential role of H_2_S-donors in restoring the HHcy-induced imbalance in the transsulfuration pathway in murine cardiomyocytes [61]. They observed that increasing concentrations of Hcy (5–100 µM) led to a significant increase in CSE gene and protein levels. In contrast, the same concentrations of the H_2_S-donors Na_2_S and GYY4137 restored normal CSE gene and protein expression levels. The effects of Hcy and H_2_S-donors on CBS were almost opposite to those observed on CSE. In fact, treatment with Hcy reduced protein levels of CBS in murine cardiomyocytes, while Na_2_S restored them. This suggests that when CBS is up-regulated, CSE is down-regulated and vice versa. A similar result was obtained in a murine model of HHcy (i.e., CBS^+/−^ mice), where CBS deficiency led to an up-regulation of *CSE* in the mouse heart, thus confirming the imbalance in the transsulfuration pathway under HHcy conditions. This is probably due to the increased activity of specificity protein-1 (SP1), an inducer of CSE, during HHcy. Accordingly, SP1 activity was reduced in murine cardiomyocytes exposed to Na_2_S. Finally, co-treatment of murine cardiomyocytes with Hcy (100 µM) and Na_2_S (30 µM) for 24 h significantly mitigated cardiac hypertrophy induced by HHcy, in terms of increased cell area and expression of molecular markers of cardiac remodeling, such as atrial-natriuretic peptide (ANP) and β-myosin heavy chain (β-MHC). This could be due, in part, to the modulation of the anti-hypertrophic miR-133a, whose expression was reduced by HHcy and up-regulated by Na_2_S [61,130].

The cardioprotective effects observed in vitro were confirmed in the study by Wang and colleagues, who demonstrated that daily administration of the sodium salt NaHS (80 µM/day, intraperitoneally, for up to 12 weeks) significantly prevented the impairment in left ventricular systolic and diastolic functions, mitochondrial alterations (i.e., enlargement of organelles), and cardiomyocytes apoptosis in rats with HHcy induced by L-Met (2 g/kg/day, dissolved in 2.5% starch, by gavage, for 18 weeks) [131]. In this experimental model, daily treatment with L-Met led to a marked decrease in CSE enzyme expression in the rat heart tissue, indicating imbalance in the transsulfuration pathway under HHcy conditions. The results obtained in the animal model of HHcy were then corroborated in primary cultures of neonatal rat cardiomyocytes, where incubation of NaHS (80 µM) for 3 days reduced mitochondrial ROS production, restored the expression of the mitochondrial NOX4 protein—a major source of oxidative stress in mitochondria—and cytochrome c in both cytoplasm and mitochondria, and preserved mitochondrial membrane potential in cells treated with Hcy (100 µM). These effects were partially or, in some cases, even completely abolished in the presence of the eNOS inhibitor N(ω)-nitro-L-arginine methyl ester (L-NAME), suggesting a potential role for this enzyme in the pharmacological properties of NaHS. Indeed, NaHS significantly prevented the HHcy-induced down-regulation of p-eNOS expression in primary rat cardiomyocytes [131].

In a rat model of HHcy induced by intraperitoneal administration of DL-Hcy (0.3 mmol/g BW during the first week to 0.6 mmol/g BW during the third week, twice a day for 3 weeks), daily treatment with an exogenous source of H_2_S (2.8 or 14 µmol/kg, intraperitoneally) during the whole period significantly restored H_2_S levels in the myocardium and reduced total Hcy concentration in plasma. In addition, H_2_S prevented the increase in MDA content in the myocardium and plasma induced by HHcy. Finally, H_2_S significantly reduced the activity of mitochondrial enzymes involved in the oxidative processes (i.e., Mn-SOD and cyclooxygenase, COX). This suggests a potential role for H_2_S in preventing the myocardial damage under HHcy conditions, probably via improvement of mitochondrial function [58].

In another work, a murine model of atherosclerosis with HHcy was created by supplying L-Met (1 g/kg dissolved in drinking water for 16 weeks) to ApoE^−/−^ mice. Daily treatment with NaHS (5.6 mg/kg, twice a day) or GYY4137 (3.6 mg/kg, twice a day), both intraperitoneally for 16 weeks, reduced blood lipid levels (i.e., total cholesterol and LDL cholesterol), serum levels of Hcy and atherosclerotic lesions in the aorta of atherosclerotic mice with HHcy. The H_2_S-donors significantly increased the gene and protein expression of the H_2_S-synthetizing enzymes CSE and 3-MST in the mouse aorta, without affecting CBS gene and protein levels. Finally, the biosynthesis of H_2_S in the aortic tissue was markedly enhanced at the end of the 16-week treatment with NaHS and GYY4137 compared to vehicle-treated mice, suggesting that the exogenous administration of H_2_S could be a valid ally for counteracting the vascular damage induced by atherosclerosis and HHcy [132]. The same experimental model has been used to unravel the mechanism responsible for the protective effects of the two H_2_S-donors in atherosclerotic vessels. NaHS and GYY4137 reversed Hcy-induced ER stress in aortic plaques by reducing the expression of protein disulphide isomerase (PDI), a redox-dependent protein and a key enzyme of protein folding, in VSMCs and endothelial cells. These effects were confirmed in cultured human aortic endothelial cells (HAECs) where, at the end of a 24 h-treatment with Hcy (200 µM), both H_2_S-donors (1 mM for 2 h) significantly reduced PDI protein expression and normalized its activity by S-sulfhydation of Cys residues [133]. This mechanism is specific to H_2_S and involves a post-translational modification that alters protein structure and function [134,135].

Many cardiometabolic disorders, including T2D, are associated with HHcy and CV dysfunction. A few years ago, HHcy was induced in a mouse model of T2D (db/db mice) by feeding animals a standard diet enriched with Met (2.0%) for 8 weeks. HHcy exacerbated the vasorelaxation impairment caused by T2D in isolated small mesenteric arteries, probably by further reducing the expression of CSE and, thus, the biosynthesis of H_2_S in the vessels. Treatment with the natural H_2_S-donor diallytrisulfide (DATS; 5 µM for 30 min) or NaHS (10–60 µM) significantly rescued oxidative stress and restored vasorelaxation through the opening of ATP-sensitive potassium (KATP) channels in isolated arteries [136]. This is a well-described mechanism responsible for the vasorelaxant effects of H_2_S and H_2_S-donors [120,137,138]. Finally, in a very recent study, the H_2_S-donor GYY4137 (150 µM up to 96 h) restored intracellular levels of H_2_S and reduced mitochondrial levels of ROS, DNA damage and mitophagy in human retinal endothelial cells concomitantly exposed to D-glucose (20 mM) and Hcy (100 µM). Deficiency of H_2_S, impaired mitochondrial function and retinal vascular damage were also confirmed in a mouse model of HHcy (CBS^+/−^ mice) and streptozotocin-induced T2D [35].

### 4.2. Brain

Blood–brain barrier is a specialized structure in which the endothelium plays a crucial role in maintaining tissue integrity and, therefore, brain function. Very recently, Yakovlev and colleagues demonstrated that daily treatment with H_2_S (source unknown) significantly prevented disruption of the blood–brain barrier in rats with prenatal HHcy. They supplemented female rats with Met (7.7 g/kg of diet) 3 weeks prior to and during pregnancy or with Met and NaHS (3 mg/kg, subcutaneously, alternating 7 days of injections with 7 days of adaptation). Treatment with NaHS significantly reduced Hcy levels and completely restored H_2_S concentration in the brains of rats. Furthermore, the H_2_S-donor markedly preserved blood–brain barrier integrity, improved mitochondrial activity, and reduced brain levels of nitrites and pro-inflammatory cytokines (i.e., interleukins—IL-1β and IL-6, and tumor necrosis factor-α—TNF-α) in the offspring [59]. This was confirmed by the improvement in developmental impairments [139] and the alleviation of motor and cognitive dysfunctions in the newborns, in part through the increased activity of antioxidant enzymes (i.e., SOD, GPx) in the brain tissue [140].

Besides maternal supplementation, daily treatment of adult rats with Hcy (0.03 µmol/g, subcutaneously, twice a day for 30 days) led to a significant increase in blood–brain barrier permeability and, therefore, in brain edema. At the same time, Hcy enhanced the expression and activity of matrix metalloproteinases (MMPs) enzymes in the cortex and hippocampus, which play a crucial role in tissue remodeling [141]. Co-treatment with NaHS (30 µmol/kg/day, intraperitoneally for 30 days) prevented all these events, indicating the pivotal role of H_2_S in the maintenance of blood–brain barrier integrity under HHcy conditions via inhibition of MMPs [60]. In the same experimental model, NaHS also prevented mitochondrial production of ROS and apoptosis, reduced nitrite levels and Mn-SOD activity, restored mitochondrial function, and decreased mitochondrial swelling in the brain of rats with HHcy [142]. In addition, NaHS treatment increased CBS and CSE activity, normalized levels of H_2_S and polysulfides in brain homogenates, and reduced the Hcy-induced production of pro-inflammatory mediators (i.e., IL-6 and TNF-α) and the expression of the inflammatory enzyme inducible nitric oxide synthase (iNOS). This further investigation of the pharmacological effects of H_2_S-donors under conditions of transsulfuration pathway imbalance provided deeper insights into the role of the vascular endothelium. Indeed, Hcy resulted in a significant increase in the expression of platelet endothelial cell adhesion molecule (PECAM) in microvessels isolated from the cortex of animals with HHcy. In contrast, microvessels from rats treated with NaHS for 30 days showed a significant decrease in PECAM expression. The sulfide salt also reversed the loss of eNOS gene and protein expression in the cortex of HHcy animals [143]. Finally, daily treatment with NaHS reduced DNA fragmentation in the cortex and hippocampus [144] and improved memory and cognitive deficits induced by Hcy [143,144]. This was due, in part, to the reversal of acetylcholinesterase activity, reduced chromatin condensation (pyknosis), and increased cytosolic levels of the antioxidant transcription factor Nrf2, which led to a reduction in oxidative stress by increasing the activity of antioxidant enzymes (i.e., GPx and catalase) and restoring the redox state in the cortex and hippocampus of animals with HHcy [145].

More recently, a murine model of HHcy was developed by adding DL-Hcy in drinking water up to a final concentration of 1.8 g/L. Supplementation ended after 10 weeks and animals were concomitantly treated with NaHS (20 µM/day, orally). In this experimental protocol, NaHS was not able to reverse the increase in plasma Hcy levels but significantly restored physiological concentrations of H_2_S in the mouse brain, which were lowered by HHcy. NaHS also increased the activity of the antioxidant enzymes SOD, catalase, and GPx, and enhanced levels of GSH. Finally, the sodium salt reduced the activity and expression of MMPs, thus confirming the results by Kumar and co-workers [60]. Worthy to note, NaHS lowered nitrite levels, thus suggesting a potential role of the vascular endothelium in the cerebroprotective effects of this H_2_S-donor. In fact, permeability of the blood–brain barrier was markedly increased in mice with HHcy, while the H_2_S-donor NaHS preserved barrier integrity [146].

The neuroprotective effects of exogenous sources of H_2_S have also been examined in vitro. For instance, the H_2_S-donor NaHS (250 µM) significantly prevented cytotoxicity, ROS production, alterations in mitochondrial membrane potential, apoptosis and changes in cell cycle in rat neuroblasts N2a co-treated with Hcy (5 mM) for 24 h [144]. In another study, pre-treatment of rat pheochromocytoma PC12 cells, often differentiated as a neuronal cell model, with the H_2_S-donor sildenafil (16 µM for 30 min) significantly prevented apoptosis and ROS production induced by Hcy (5 mM for 24 h), probably via activation of the atheroprotective paraoxonase-1 (PON-1) enzyme [147,148].

### 4.3. Kidney

Vascular endothelium plays a crucial role in kidney function, and endothelial dysfunction is recognized as a key feature of kidney disease [149,150]. Therefore, any impairment in vascular function, such as under HHcy conditions, can contribute to renal damage. Genetically-induced HHcy (CBS^+/−^ mice) was associated with a marked reduction in plasma levels of H_2_S, glomerular filtration rate (GFR) and renal cortical blood flow. Treatment with the sodium salt NaHS (30 µM/day for 8 weeks in drinking water) significantly restored renal cortical vascularity, probably by increasing eNOS activity in the kidney. Furthermore, NaHS prevented the increase in MMPs expression and collagen deposition in the kidney of CBS^+/−^ mice, and reduced proliferation of cultured VSMCs and renal artery explants exposed to Hcy (75 µM) [57]. These findings confirm the endothelial-mediated pharmacological effects of H_2_S in the renal tissue, supporting the hypothesis of an “invisible thread” that links different districts and underlies both HHcy-induced and H_2_S-reversed organ damage, namely vascular endothelium. Treatment of isolated renal arteries with Hcy (75 µM for 48 h) in the presence of NaHS (30 µM) led to a significant improvement in vasorelaxation. Worthy of note, this effect was completely abolished in the presence of the eNOS inhibitor L-NAME, further indicating the central role of the endothelium in the pharmacology of H_2_S.

In the same experimental model, daily treatment with NaHS (30 µM/day for 8 weeks in drinking water) significantly prevented the increase in plasma levels of Hcy, enhanced H_2_S bioavailability, reduced glomerular oxidative stress and cell death, increased the ratio of reduced to oxidized GSH (GSH/GSSG) in cortical tissue, and normalized MMPs activity in the kidney of CBS^+/−^ mice. These events ameliorated Hcy-induced renopathy, opening the way for further studies on H_2_S pharmacology [151]. Similarly, the H_2_S-donor GYY4137 (250 µM) significantly reduced the activity of MMPs in mesangial cells exposed to Hcy (50 µM for 30 min). In addition, in this series of experiments in vitro, GYY4137 counteracted apoptosis, reduced oxidative stress, mitigated mitochondrial dysfunction, and prevented the increase in the expression of collagen I and fibronectin in mesangial cells treated with Hcy (50 µM for 24 h), suggesting a potential role for H_2_S in attenuating tissue remodeling processes under HHcy [152].

### 4.4. Liver

The liver is a highly perfused organ that may be particularly susceptible to HHcy-mediated toxicity. However, very few studies examined the effects of H_2_S-donors against HHcy-induced hepatic damage. The natural H_2_S-donor sulforaphane (from broccoli, *Brassica oleracea* L.) significantly attenuated oxidative stress in human hepatocytes exposed to high concentrations of Hcy. In this study, pre-incubation of sulforaphane (1–20 µM for 24 h) preserved cell viability and reduced ROS production induced by treatment with Hcy (10 mM for 24 h and 6 h, respectively). Sulforaphane also reduced the expression of MDA and enhanced that of antioxidant enzymes concentration-dependently, in part by promoting the phosphorylation of the antioxidant transcription factor Nrf2 [153]. In another study, daily treatment with NaHS (10 mg/kg/day, intraperitoneally) restored serum and hepatic levels of Hcy in mice with diet-induced HHcy (i.e., mice fed for 17 weeks with chow containing 2.5% Met). In addition, the H_2_S-donor alleviated liver injury by significantly reducing transaminase concentration, mRNA expression of inflammatory genes, and autophagy in livers from HHcy mice. The latter effect was probably due to S-sulfhydation and activation of the autophagy regulator serum and glucocorticoid-regulated kinase 1 (SGK1) at Cys244 and Cys282 induced by NaHS (200 µM for 24 h), as demonstrated on cultured HepG2 hepatic cells. Finally, NaHS reduced oxidative stress and lipid deposition in vitro, further confirming the beneficial effects against liver injury observed in the in vivo model of HHcy [154].

The pharmacological effects of H_2_S-donors under HHcy conditions are summarized in Table 2.

## 5. Conclusions

This review examined the current literature on the potential pharmacological effects of upstream modulators of the transsulfuration pathway (i.e., folic acid), downstream products of this pathway (i.e., cysteine, cystine, serine, taurine), and H_2_S-donors under HHcy conditions. The findings offer a comprehensive overview of both “traditional” (i.e., folic acid) and “emerging” (i.e., transsulfuration pathway products and H_2_S-donors) pharmacological strategies, which may help restore transsulfuration balance by normalizing physiological levels of Hcy and, in turn, prevent HHcy-induced organ and tissue dysfunction. Beyond their Hcy-lowering effects, a common underlying mechanism may involve mitochondrial protection and the preservation of endothelial barrier integrity. This could not only support cellular function and homeostasis, but also limit the spread of ROS, inflammatory mediators, and Hcy itself to surrounding tissues, thus reducing HHcy-associated complications in the CV system, brain, kidneys, and liver.

In conclusion, this review opens new perspectives for the treatment of HHcy and the prevention of related disorders through the use of folic acid, cysteine, N-acetylcysteine, serine, taurine, and H_2_S-donors. However, a major limitation of the current evidence on H_2_S-donors is the large use of “fast” H_2_S-releasing compounds in both in vitro and in vivo studies, which could lead to misleading, non-generalizable results, and potential toxic effects. Therefore, further studies are needed to clarify the therapeutic potential and safety profile of these compounds in HHcy conditions.

## Figures and Tables

**Table 1:** Summary of the key findings from original preclinical and clinical studies focusing on treatment/supplementation with upstream modulators (i.e., folic acid) and downstream products (i.e., cysteine, N-acetylcysteine, serine, and taurine) of the transsulfuration pathway under HHcy conditions. Abbreviations: BH4, tetrahydrobiopterin; BW, body weight; CBS, cystathionine β-synthase; CDO, cysteine dioxygenase; CSAD, cysteine sulfinic acid decarboxylase; Cys, cysteine; FMD, flow-mediated dilation; GOT1, glutamic oxaloacetic transaminase 1; HCU, homocystinuria; Hcy, homocysteine; HHcy, hyperhomocysteinemia; HcyT, homocysteine thiolactone; HUVECs, human umbilical vein endothelial cells; i.p., intraperitoneally; i.v., intravenously; LDH, lactate dehydrogenase; Met, methionine; Mn-SOD, Mn-superoxide dismutase; NAC, N-acetylcysteine; NO, nitric oxide; ROS, reactive oxygen species; SHRs, spontaneously hypertensive rats; VSMCs, vascular smooth muscle cells. Symbols: ↑ increase; ↓ decrease.

First Author, Year	Preclinical Model/Subjects	Treatment (Concentration/Dose)	Duration	Main Findings
Preclinical studies				
Cao, 2021 [85]	SHRs with Hcy-induced HHcy	Folic acid (0.4 mg/kg/day, orally)	6 weeks	↓ Interstitial and perivascular collagen deposition in cardiomyocytes ↓ Diastolic dysfunction
Chang, 2004 [102]	VSMCs exposed to HHcy	Taurine (5, 10, and 20 mM)	12 h	↓ LDH and ROS production ↑ Mn-SOD and catalase activity
Chang, 2004 [104]	Rat mitochondria exposed to HHcy	Taurine (5,10, and 20 µM)	-	↓ Ca^2+^ uptake inhibition
Chang, 2004 [105]	Rats with DL-Met-induced HHcy	L-Taurine (1% in the chow)	6 weeks	↓ ROS production ↑ Ca^2+^ fluxes in myocardial mitochondria
Cui, 2018 [82]	HUVECs exposed to HHcy	Folic acid (0-1 µM)	48 h	↑ Cell viability ↓ Apoptosis
Fukada, 2006 [94]	Rats with Met-induced HHcy	Serine (0.5%, 1%, or 2% in the chow; 100, 200, 300, or 500 mg/kg/day, i.p.)	10 days	↓ Hcy levels
Jiang, 2014 [93]	CBS-deficient HCU mice	Cys (1.5 mg/mL in drinking water)	1 week	Normalization of hepatic CDO levels
Kondakçı, 2017 [96]	Rats with HcyT-induced HHcy	NAC (1 g/kg/day, unknown route of administration)	6 weeks	↓ Serum Hcy ↓ Hepatic and renal ROS
Lee, 2005 [84]	Rats with Hcy-induced HHcy	Folic acid (8 mg per kg of chow)	8 weeks	↓ Vascular damage ↓ Serum Hcy levels
Maclean, 2021 [107]	CBS-deficient HCU mice	Taurine (20 mg/mL in drinking water)	1 week	Normalization of hepatic expression levels of CDO, CSAD, and GOT1
Nonaka, 2009 [101]	VSMCs exposed to HHcy	Taurine (10 mM)	30 min	↓ Oxidative stress in the vascular wall
Okawa, 2007 [92]	Rats with HHcy induced by low-protein diet	Cys (0.3% or 0.6% in the chow)	14 days	↓ HHcy
Wu, 2024 [97]	HUVECs exposed to HHcy	NAC (5 mM)	48 h	↓ Morphofunctional alterations in the vascular endothelium
Yalçinkaya, 2009 [106]	Rats with HHcy induced by high-Met diet	Taurine (1.5% *w*/*v* in drinking water)	6 months	↓ Oxidative stress ↓ Nitrosative stress ↓ Apoptosis ↓ Hepatic necrosis
Zhang, 2014 [83]	HUVECs exposed to HHcy	Folic acid (5–10 nM)	12 h	↑ NO production ↑ BH4 levels
Zhang, 2017 [103]	Rat cardiomyoblasts exposed to HHcy	Taurine (40 mM)	60 min	↓ Apoptosis
Clinical Studies				
Hildebrandt, 2015 [98]	Hyperlipidemic and normolipidemic men	NAC (1.8 g/day, orally)	4 weeks	↓ Plasma Hcy concentrations ↓ Systolic blood pressure
Scholze, 2004 [100]	Patients with end-stage renal failure	NAC (5 g, i.v.)	During hemodialysis (4 h)	↓ Plasma Hcy levels ↑ Endothelial function
Thaha, 2006 [99]	Patients with end-stage renal failure	NAC (5 g, i.v.)	During hemodialysis (4 h)	↓ Plasma Hcy levels ↓ Blood pressure ↓ Pulse pressure
Van Hove, 2019 [108]	Patients with inherited CBS-deficient HCU	Taurine (75 mg/kg, orally)	4 days	↑ FMD
Verhoef, 2004 [95]	Healthy men who ingested low-protein diet supplemented with Met (once, at breakfast)	Serine (60.6 mg/kg/BW) and cystine (12.3 mg/kg/BW), both in the diet	Once (at breakfast)	↓ Plasma Hcy levels
Vianna, 2007 [90]	Patients with end-stage renal disease	Folic acid (10 mg, orally)	2 years	↓ Blood Hcy levels ↓ Intima-media wall thickness

**Table 2:** Summary of key findings from preclinical studies focusing on treatment with H_2_S-donors under HHcy conditions. Abbreviations: 3-MST, 3-mercaptopyruvate sulfurtransferase; CBS, cystathionine β-synthase; COX, cyclooxygenase; CSE, cystathionine γ-liase; DATS, diallytrisulfide; eNOS, endothelial nitric oxide synthase; ER, endoplasmic reticulum; GPx, glutathione peroxidase; GSH, reduced glutathione; GSSG, oxidized glutathione; H_2_S, hydrogen sulfide; Hcy, homocysteine; HHcy, hyperhomocysteinemia; HAECs, human aortic endothelial cells; i.p., intraperitoneally; iNOS, inducible nitric oxide synthase; Met, methionine; MMPs, matrix metalloproteinases; Mn-SOD, Mn-superoxide dismutase; PDI, protein disulphide isomerase; PECAM, platelet endothelial cell adhesion molecule; ROS, reactive oxygen species; SP1, specificity protein-1; VSMCs, vascular smooth muscle cells. Symbols: ↑ increase; ↓ decrease; * gene and protein.

First Author, Year	Preclinical Model	Treatment (Concentration/Dose)	Duration	Main Findings
Chang, 2008 [58]	Rats with DL-Hcy-induced HHcy	Unknown source of H_2_S (2.8 or 14 µmol/kg/day, i.p.)	3 weeks	↑ H_2_S levels in myocardium ↓ Plasma Hcy levels ↓ Mn-SOD and COX activity
Cheng, 2018 [136]	Small mesenteric arteries of db/db mice with high-Met diet-induced HHcy	DATS (5 µM) or NaHS (10–60 µM)	30 min	↓ Oxidative stress ↑ Vasorelaxation
Fan, 2019 [132]	ApoE^−/−^ atherosclerotic mice with L-Met-induced HHcy	NaHS (5.6 mg/kg twice a day, i.p.) or GYY4137 (3.6 mg/kg twice a day, i.p.)	16 weeks	↓ Blood lipid levels ↓ Serum Hcy ↓ Atherosclerotic lesions in aortic tissue ↑ CSE and 3-MST expression * in aortic tissue ↑ H_2_S levels in aortic tissue
He, 2014 [153]	Human hepatocytes exposed to HHcy	Sulforaphane (1–20 µM)	24 h	↓ ROS production
Jiang, 2021 [133]	ApoE^−/−^ atherosclerotic mice with L-Met-induced HHcy	NaHS (5.6 mg/kg twice a day, i.p.) or GYY4137 (3.6 mg/kg twice a day, i.p.)	16 weeks	↓ ER stress in aortic plaques
Jiang, 2021 [133]	HAECs exposed to HHcy	NaHS (1 mM) or GYY4137 (1 mM)	2 h	↓ PDI expression
Kesherwani, 2015 [130]	Murine cardiomyocytes exposed to HHcy	Na_2_S (30 µM)	24 h	↓ Cardiac hypertrophy
Kumar, 2018a [144]	N2a cells exposed to HHcy	NaHS (250 µM)	24 h	Prevention of cytotoxicity, ROS production, alterations in mitochondrial membrane potential, apoptosis, and changes in cell cycle
Kumar, 2018a [144]	Rats with Hcy-induced HHcy	NaHS (30 µmol/kg/day, i.p.)	30 days	↓ DNA fragmentation in the cortex and hippocampus ↑ Memory and cognitive deficits
Kumar, 2018b [145]	Rats with Hcy-induced HHcy	NaHS (30 µmol/kg/day, i.p.)	30 days	Reverse of acetylcholinesterase activity ↓ Chromatin condensation ↓ Oxidative stress in the cortex and hippocampus
Kumar, 2019 [143]	Rats with Hcy-induced HHcy	NaHS (30 µmol/kg/day, i.p.)	30 days	↑ CBS and CSE activity Normalization of H_2_S and polysulfides levels in the brain ↓ Pro-inflammatory mediators ↓ iNOS expression ↓ PECAM expression in cortical microvessels Reversal of eNOS expression* in the cortex
Kumar, 2020 [142]	Rats with Hcy-induced HHcy	NaHS (30 µmol/kg/day, i.p.)	30 days	Prevention of apoptosis and ROS production ↓ Mitochondrial swelling in the brain ↓ Nitrite levels ↓ Mn-SOD activity
Kumar, 2022 [60]	Rats with Hcy-induced HHcy	NaHS (30 µmol/kg/day, i.p.)	30 days	Maintenance of blood–brain barrier integrity via inhibition of MMPs
Majumder, 2019 [152]	Mesangial cells exposed to HHcy	GYY4137 (250 µM)	24 h	↓ MMPs activity ↓ Apoptosis ↓ Oxidative stress ↓ Mitochondrial dysfunction Prevention of collagen I and fibronectin expression
Malaviya, 2024 [35]	Human retinal endothelial cells exposed to HHcy	GYY4137 (150 µM)	96 h	↑ H_2_S intracellular levels ↓ ROS mitochondrial levels ↓ DNA damage ↓ Mitophagy
Nath, 2019 [146]	Mice with DL-Hcy-induced HHcy	NaHS (20 µM/day, orally)	10 weeks	Restoration of H_2_S physiological concentrations in the brain ↑ Antioxidant enzymes activity ↑ GSH levels ↓ MMPs ↓ Nitrite levels Maintenance of blood–brain barrier integrity
Nandi, 2017 [61]	Murine cardiomyocytes exposed to HHcy	Na_2_S (5–100 µM) or GYY4137 (5–100 µM)	24 h	↓ CSE expression* ↑ CBS expression ↓ SP1 activity
Pushpakumar, 2019 [57]	CBS^+/−^ mice	NaHS (30 µM/day, orally)	8 weeks	Restoration of renal cortical vascularity Prevention of the increase in MMPs expression and collagen deposition in the kidney
Pushpakumar, 2019 [57]	VSMCs exposed to HHcy	NaHS (30 µM, orally)	48 h	↓ Proliferation
Sen, 2009 [151]	CBS^+/−^ mice	NaHS (30 µM/day orally)	8 weeks	Prevention of the increase in plasma Hcy ↑ H_2_S bioavailability ↓ Glomerular oxidative stress ↓ Cell death ↑ GSH/GSSG in cortical tissue Normalization of MMPs activity in the kidney
Tang, 2013 [147]	PC12 rat cells exposed to HHcy	Sildenafil (16 µM)	30 min	Prevention of apoptosis and ROS production
Wang, 2015 [131]	Rats with L-Met-induced HHcy	NaHS (80 µM/day, i.p.)	12 weeks	↓ Cardiac impairment ↓ Mitochondrial alterations ↓ Cardiomyocytes apoptosis
Wang, 2015 [131]	Neonatal rat cardiomyocytes exposed to HHcy	NaHS (80 µM)	3 days	↓ ROS production
Yakovleva, 2018 [139]	Rat offspring with prenatal HHcy induced by Met	NaHS (3 mg/kg, subcutaneously, alternating 7 days of injections with 7 days of adaptation)	3 weeks prior to and during pregnancy	Improvement in developmental impairments
Yakovleva, 2020 [140]	Rat offspring with prenatal HHcy induced by Met	NaHS (3 mg/kg, subcutaneously, alternating 7 days of injections with 7 days of adaptation)	3 weeks prior to and during pregnancy	Alleviation of motor and cognitive dysfunctions ↑ SOD and GPx activity in the brain
Yakovlev, 2024 [59]	Rat offspring with prenatal HHcy induced by Met	NaHS (3 mg/kg, subcutaneously, alternating 7 days of injections with 7 days of adaptation)	3 weeks prior to and during pregnancy	↓ Hcy levels Restoration of H_2_S levels in the brain Preservation of blood–brain barrier integrity, ↑ mitochondrial activity, and ↓ brain levels of nitrites and pro-inflammatory cytokines
Zhu, 2024 [154]	Mice with HHcy induced by high-Met diet	NaHS (10 mg/kg/day, i.p.)	17 weeks	↓ Liver injury
Zhu, 2024 [154]	HepG2 hepatic cells exposed to HHcy	NaHS (200 µM)	24 h	↓ Autophagy ↓ Oxidative stress ↓ Lipid deposition

## Data Availability

Not applicable.

## Abbreviations

The following abbreviations are used in this manuscript:

3-MST	3-mercaptopyruvate sulfurtransferase
ANP	Atrial-natriuretic peptide
ARE	Antioxidant response element
ATP	Adenosine triphosphate
BAX	BCL-2-like protein 4
BCL2	B-cell lymphoma 2
BHMT	Betaine-homocysteine methyltransferase
BW	Body weight
CASP3	Caspase-3
CASP8	Caspase-8
CAT	Cysteine aminotransferase
CBS	Cystathionine β-synthase
CHF	Chronic heart failure
CDO	Cysteine dioxygenase
CO	Carbon monoxide
COX	Cyclooxygenase
CSAD	Cysteine sulfinic acid decarboxylase
CSE	Cystathionine γ-liase
CV	Cardiovascular
DATS	Diallytrisulfide
EC-SOD	Extracellular superoxide dismutase
eNOS	Endothelial nitric oxide synthase
ER	Endoplasmic reticulum
FMD	Flow-mediated dilation
GFR	Glomerular filtration rate
GOT1	Glutamic oxaloacetic transaminase 1
GPx	Glutathione peroxidase
GS	Glutathione synthase
HAECs	Human aortic endothelial cells
HCU	Homocystinuria
HcyT	Homocysteine thiolactone
HDL	High-density lipoprotein
HFpEF	Heart failure with preserved ejection fraction
HHcy	Hyperhomocysteinemia
HO-1	Heme oxygenase-1
HTAU-DH	Hypotaurine dehydrogenase
HUVECs	Human umbilical vein endothelial cells
IL	Interleukin
iNOS	Inducible nitric oxide synthase
KATP channels	ATP-sensitive potassium channels
Keap1	Kelch-like ECH-associated protein 1
L-NAME	N(ω)-nitro-L-arginine methyl ester
LDH	Lactate dehydrogenase
LDL	Low-density lipoprotein
MMPs	Matrix metalloproteinases
Mn-SOD	Mn-superoxide dismutase
MS	Methionine synthase
MTHFR	Methylenetetrahydrofolate reductase
NAC	N-acetylcysteine
NF-κB	Nuclear factor kappa-light-chain-enhancer of activated B cells
NFS1	Mitochondrial cysteine desulfurase
NO	Nitric oxide
NOX	NADPH oxidase
Nrf2	Nuclear factor erythroid 2-related factor 2
OPA3	Optic atrophy 3
OTUB1	OTU domain-containing ubiquitin aldehyde-binding protein 1
PDI	Protein disulphide isomerase
PECAM	Platelet endothelial cell adhesion molecule
PON-1	Paraoxonase-1
RNS	Reactive nitrogen species
ROS	Reactive oxygen species
SGK1	Serum and glucocorticoid-regulated kinase 1
SHRs	Spontaneously hypertensive rats
SIRT1	Sirtuin isoform 1
SOD	Superoxide dismutase
SP1	Specificity protein-1
T2D	Type 2 diabetes
THF	Tetrahydrofolate
TNF-α	Tumor necrosis factor-α
TP53	Tumor protein 53
VSMCs	Vascular smooth muscle cells
Xc^−^	Cystine/glutamate antiporter
xCT	Cystine/glutamate antiporter light chain subunit
XO	Xanthine oxidase
β-MHC	β-myosin heavy chain
γ-GCS	γ-glutamyl cysteine synthetase
**Glossary of Common Metabolite Terms**	
BH4	Tetrahydrobiopterin
Cys	Cysteine
DMG	Dimethylglycine
GSH	Reduced glutathione
GSSG	Oxidized glutathione
Hcy	Homocysteine
MDA	Malondialdehyde
Met	Methionine
SAH	S-adenosyl homocysteine
SAM	S-adenosyl methionine
THF	Tetrahydrofolate
γ-Glu-Cys	γ-glutamyl cysteine
